# Swallowing Guidance with FEES May Alleviate Symptoms in Functional Dysphagia

**DOI:** 10.1007/s00455-025-10869-5

**Published:** 2025-09-12

**Authors:** Jonna Kuuskoski, Jami Rekola, Harri Sintonen, Leena-Maija Aaltonen, Pia Järvenpää

**Affiliations:** 1 Department of Otorhinolaryngology – Head and Neck Surgery, Turku University Hospital and University of Turku, P.O. Box 52, Turku, 20521 Finland; 2 Department of Public Health, University of Helsinki, Helsinki, Finland; 3 Department of Otorhinolaryngology – Head and Neck Surgery, Helsinki University Hospital and University of Helsinki, Helsinki, Finland

**Keywords:** Functional dysphagia, Dysphagia symptoms, Dysphagia evaluation, Fees, Swallowing guidance

## Abstract

Dysphagia is a common concern. In an outpatient phoniatric and otorhinolaryngology clinic, approximately one fourth of dysphagia patients are classified as having non-organic, or functional dysphagia. This study aimed to evaluate symptom severity, health-related quality of life (HRQoL) and disability among dysphagia patients. Additionally, it focused on assessing the impact of flexible endoscopic evaluation of swallowing (FEES) and swallowing guidance particularly for patients with functional dysphagia. We recruited 60 consecutive dysphagia patients from our phoniatrics clinic. They completed the Eating Assessment Tool (EAT-10), the 15D Health-related Quality of Life Instrument and the World Health Organization Disability Assessment Schedule (WHODAS 2.0) questionnaires before their appointments. We performed FEES and gave swallowing guidance. One month later, the patients repeated the EAT-10, 15D, and WHODAS 2.0. At one-month follow-up, the EAT-10 scores of all 60 dysphagia patients (median age 65, range 18–89 years, 70% female), and the functional dysphagia patients (*n* = 15, 25%) had decreased significantly (*p* = 0.020, *p* = 0.029, respectively). Although the changes in the 15D and WHODAS 2.0 scores were insignificant, the score of WHODAS 2.0 item D3.3 concerning eating had decreased significantly in the functional dysphagia group (*p* = 0.020). Comparison of the whole dysphagia patient cohort to an age- and gender-standardized sample of the general population revealed significant differences in 15D total scores (*p* < 0.001) and 12 of the 15 dimensions. Dysphagia seems to significantly diminish patients’ HRQoL in comparison to that of the general population. A short FEES intervention with swallowing guidance may alleviate symptoms, especially in functional dysphagia.

## Introduction

Dysphagia is a common concern, and it is important to determine whether it is a benign sensation or a sign of a serious disease. Many diseases are associated with dysphagia, and it can have a significant impact on a patient’s life [1]. If untreated, it can at worst be life-threatening due to dehydration, malnutrition, and aspiration. On the other hand, being a complex series of coordinated movements regulated e.g. by the parasympathetic vagus nerve, swallowing is vulnerable to benign alterations, disturbances, and symptoms with no underlying organic disorder [2]. This type of ‘non-organic dysphagia’ has previously been referred to as medically unexplained dysphagia, swallowing phobia, psychogenic dysphagia, or phagophobia [2]. However, the preferred term is functional dysphagia, which falls under the broader category of functional, persistent somatic, or persistent physical symptom disorders [3]. It is also important not to confuse dysphagia with globus symptom, the sensation of a lump in the throat that is often alleviated by swallowing food or liquid [4]. Regardless of its etiology, a swallowing problem can significantly affect an individuals’ self-esteem, socialization, and quality of life [1, 5].

In our experience, the prevalence of functional dysphagia seems to be relatively high. In our earlier study of a cohort of an unselected dysphagia outpatient population, 26.5% of the patients were classified as functional [6]. Patients with functional symptoms have shown to value a personalized approach in which a physician takes personal circumstances into account, and examines and communicates with the patient properly, treating them as equal individuals [7]. In the study by Järvenpää et al., almost half of the patients with unspecific dysphagia symptoms (most of which could be classified as functional dysphagia), were asymptomatic, or their symptoms were relieved after a three-year follow-up without any specific treatment [8]. Moreover, a recent study showed that as much as 43% of the patients did not attend any swallow therapy appointments regardless of socioeconomic or demographic factors, or dysphagia etiology [9]. Thus, the information and guidance given at the initial appointment seems to have great importance.

This prospective study aimed to assess symptom severity, health-related quality of life (HRQoL), and disability of the dysphagia patient population referred to our phoniatrics outpatient clinic. After flexible endoscopic evaluation of swallowing (FEES), a physician and a speech and language pathologist (SLP) provided each patient with information and swallowing guidance, including video explanations. We then evaluated changes in symptom severity, HRQoL, and disability over a one-month follow-up period. Symptom severity was measured using the Eating Assessment tool, EAT-10 [10], HRQoL using the 15D Health-related Quality of Life Instrument [11], and the disability using the 36-item version of the World Health Organization Disability Assessment Schedule, WHODAS 2.0 [12]. The study focused specifically on patients with functional dysphagia.

## Methods

We recruited 60 consecutive adult patients who had been referred to the Turku University Hospital phoniatrics outpatient clinic due to a complaint of dysphagia from primary health care or other medical disciplines. We excluded patients with severe cognitive or psychiatric illnesses or if they were unable to complete the questionnaires independently (writing assistance was accepted). Information on age, gender, body mass index (BMI), diagnosed diseases, previously performed surgeries, medication, smoking habits, and alcohol consumption were collected. We evaluated the etiology of dysphagia from medical records and examinations.

The patients filled in the Finnish version of the EAT-10 to provide their subjective evaluation of dysphagia symptoms and symptom severity. HRQoL was assessed using the 15D, and disability using the 36-item version of WHODAS 2.0 (self-administered). Each patient completed the questionnaires just before the outpatient appointment using a paper and pencil or did the web-based survey, provided by Research Electronic Data Capture (REDCap), according to their preference. For objective evaluation of dysphagia, we performed FEES and the simplified 100 ml Water Swallow test (sWST), in which only coughing and the number of swallows are observed [13, 14]. The patients filled in the follow-up questionnaires one month later. Reminders were emailed to them three times if they had not responded to the follow-up questionnaires after one month.

### Questionnaires

#### EAT-10

The 10-item Eating Assessment Tool (EAT-10) is one of the most widely used tools for evaluating dysphagia [10]. It has been translated into multiple languages, including Finnish, and validated across diverse populations. Currently, it is distributed by the Nestlé Nutrition Institute (© Société des Produits Nestlé SA 2009). EAT-10 comprises ten items, each rated on a five-point Likert scale, ranging from no difficulty (0 points) to severe difficulty (4 points). The total score is calculated as the sum score of all item scores, producing a range of 0 to 40 [10]. For this study, we used the Finnish version of the EAT-10 [6]. In the one-month follow-up EAT-10 questionnaire, we added an item to assess subjective symptom changes over the past 30 days, scored as follows: 0 = no symptoms, 1 = fewer symptoms, 2 = symptoms unchanged, 3 = slightly more symptoms, 4 = many more symptoms.

#### 15D

The 15D is a generic, patient-reported measure of HRQoL, covering15 dimensions: mobility, vision, hearing, breathing, sleeping, eating, speech, excretion, usual activities, mental function, discomfort and symptoms, depression, distress, vitality, and sexual activity [11]. Each dimension is rated on a five-point Likert scale, where 1 represents the best possible situation and 5 the worst. A single index score, or 15D score, is calculated to represent overall HRQoL on a 0–1 scale, with 1 indicating full health and 0 representing death. Dimension-specific scores, reflecting levels from no problems (= 1) to death(= 0), are derived from population-based utility weights. Mean scores for each dimension are used to draw 15D profiles for groups [11]. The minimal clinically important difference in the overall 15D score is reported as 0.015 [15]. Normative values, matched by age and gender, were obtained from the Finnish National Health 2011 Survey [16].

In dysphagia research, the 15D has previously been used only with specific patient groups, including those with dysphagia following cervical anterior decompression surgery and patients experiencing globus symptoms [17, 18].

#### WHODAS 2.0

WHODAS 2.0, developed by the World Health Organization (WHO), is a standardized tool for measuring health and disability across six life domains: (1) cognition, (2) mobility, (3) self-care, (4) getting along, (5.1) life activities, (5.2) work and studying, and 6) participation [12]. There are three versions of WHODAS 2.0: a 36-item version, a12-item version, and a 12 + 24 item version, with the 36-item version providing the most detailed assessment. Each item is rated on a five-point Likert scale, (none = 0, mild = 1, moderate = 2, severe = 3, and extreme = 4), with scores summed by domain and overall for a total simple score. Complex scoring, based on item-response theory, weights and codes each item to produce domain scores and a total score on a 0–100 scale, where 0 represents no disability and 100 represents full disability [12].

In this study, we used the Finnish 36-item, self-administered version of WHODAS 2.0 [19]. Simple scoring was applied to compare baseline and one-month follow-up results, while complex scoring was used to benchmark the patient cohort against normative values from the Swedish population, as Finnish population norms for WHODAS 2.0 are not yet available [20]. Given Sweden’s close geographic and cultural proximity as a fellow Nordic country, Swedish norms are assumed to approximate Finnish values.

WHODAS 2.0 is widely applicable across diagnostic groups, particularly in psychiatry, geriatrics, neurology, disability and rehabilitation, health sciences, and epidemiology [21]. In dysphagia research, WHODAS 2.0 has been used to assess disability among survivors of head and neck cancer and patients with corrosive esophageal stricture [22, 23].

### FEES and Swallowing Guidance During Outpatient Appointment

The outpatient appointments at Turku University Hospital Phoniatrics Clinic lasted 90 min. with both a physician (specialist in otolaryngology and phoniatrics) and a speech-language pathologist (SLP) present. The patient was interviewed, and the FEES was performed. Before evaluating swallowing, the physician and the SLP jointly assessed velopharyngeal closure, rated possible saliva residues in the vallecula and pyriform sinuses, and evaluated tongue movement, pharyngeal wall movement, and vocal cord closure. The boluses were dyed with food coloring for better visualization, and the textures used in the FEES included liquid (water), nectar (blueberry soup), semi-solid (fruit puree), and solid (cookie). The examination began with small boluses (half a teaspoon) and progressed to larger ones (a tablespoon). Pharyngeal residues were scored using the Yale Pharyngeal Residue Severity Scale [24], while possible penetration or aspiration was assessed using the Penetration-Aspiration Scale (PAS) [25], and the Dysphagia Outcome Severity Scale (DOSS) was used for final evaluation [26].

After the FEES, we used the simplified version of the 100 ml Water Swallow Test (sWST), in which the patient is asked to drink 100 ml of water continuously. They passed the test if they did not stop their drinking, did not cough while drinking or one minute after drinking, and were able to finish the 100 ml in less than five swallows [14].

After the examination, the physician showed the FEES recordings to the patient and explained swallowing anatomy and physiology using illustrations if needed. Any disturbances in swallowing were highlighted in the videos. The most bothersome symptom was identified, and possible factors interfering with normal swallowing were detected. Facilitating factors for swallowing were also identified and explained. The patients were advised to avoid any potential avoidance behaviors, and normal, social eating was encouraged, provided no safety risks were observed during the swallowing examination. Any fears, anxieties, or distorted thoughts about swallowing were addressed, and both the physician and the SLP worked to alleviate them. Instructions were given to humidify and lubricate the nose and throat if mucosal dryness was identified. Instructions to modify swallowing speed, bolus size and/or consistency were also provided if necessary. Postural changes or swallowing maneuvers, such as the supraglottic swallow or effortful swallow, were introduced with the FEES biofeedback if considered potentially effective. If needed, chewing muscle or head and neck massage was recommended, along with instructions for self-treatment to alleviate muscle tightness. Swallowing exercises or techniques such as thermal or electrical stimulation were not included. All instructions were also provided to the patients in written form. This type of intervention is called *swallowing guidance*, which differs from swallowing therapy.

The etiology of dysphagia was determined based on findings from the outpatient appointment and any additional assessments, such as neurology consultations, videofluoroscopy, or esophageal examinations (including esophagogastroscopy, multichannel intraluminal impedance and pH monitoring, or high-resolution manometry). We categorized dysphagia etiologies into the following groups: functional, globus, dry mouth/throat, neurological, esophageal (reflux, motility disorder, esophagitis), compression (e.g., cervical osteophyte), presbyphagia, and head and neck or esophageal cancer. Functional dysphagia is defined as swallowing difficulty without a structural, neurological, or other identifiable physical cause in the digestive tract.

For the FEES, we used a 3.9 mm–2.6 mm videoendoscope (Olympus HD ENF-VH or -V3), an Olympus Evis Exera III HDTV video processor CV-190 and CLL-S1 light source, (Olympus Europa, Hamburg, Germany). All the recordings were made by the rpSzene Software (rpSzene, Rheder, Hamburg, Germany).

#### Ethical Considerations

Before participating, the recruited patients received both verbal and written information about the study and were asked to provide their written consent to participation. The Ethics Committee of the Hospital District of Southwest Finland approved the study protocol, and permission for the research was obtained from the same district. This study was carried out in compliance with the principles outlined in the Declaration of Helsinki (The World Medical Association 2013).

### Sample Size and Statistic Methods

The sample size was determined by estimating various means and standard deviations (SD) for changes in the EAT-10 total score between baseline and one-month follow-up, using the Wilcoxon Signed Rank Test. Assuming an expected decrease of two points in the EAT-10 total score, a sample size of 54 participants was calculated. With an anticipated 10% dropout rate, the required sample size was set at 60 participants.

HRQoL was assessed by comparing baseline 15D scores to an age- and gender-matched sample from the general Finnish population, with differences evaluated using an independent samples t-test. One-way ANOVA with Bonferroni corrections was used to post hoc comparison of 15D means between different diagnostic groups. Differences in WHODAS 2.0 domain scores and total scores between the initial appointment and the one-month follow-up were analyzed with the Wilcoxon Signed Rank Test. Correlation between age and EAT-10 total scores was assessed with Spearman’s rho. The effect of gender on EAT-10 scores was tested with the Mann-Whitney U test, while the impact on subjective symptom change was evaluated using the Chi-square test. Differences in age and gender distribution between participants who returned follow-up questionnaires and those who did not were also assessed using the Mann-Whitney U test and the Chi-square test, respectively. P-values < 0.05 were considered statistically significant.

We consulted an experienced statistician for the statistical analyses, performed with the IBM SPSS Statistics for Windows (version 29.0; IBM Corp., Armonk, NY, USA).

## Results

Sixty consecutive patients were recruited from October 2021 to September 2023 at the Turku University Hospital Phoniatrics outpatient clinic. Recruitment was extended due to the COVID-19 pandemic and work arrangement issues. The median age of all the participants was 65 years (range 18–89 years; interquartile range (IQR) 45–74 years), with 70% being female. The median BMI was 27.5 (range 16.9–48.3, IQR 23.1–30.9). In the functional dysphagia group (*n* = 15, 25.0%), the median age was significantly lower (*p* = 0.007) than in the entire cohort, at 42 years (range 18–79, IQR 22–59) and 93.3% (*n* = 14) were female. The median BMI was the same in the functional dysphagia group as in the total cohort, at 27.5 (range 16.9–40.7, IQR 21.7–32.3). Dysphagia etiologies are detailed in Table 1. The median Dysphagia Outcome Severity Scale (DOSS) score was 7 (IQR 6–7) and did not differ significantly across etiology groups.

**Table 1:** Etiology of dysphagia among 60 patients referred to phoniatrics outpatient clinic

Etiology of dysphagia	*n* (%)
Functional	15 (25.0%)
Globus	10 (16.7%)
Dry mouth/throat	9 (15.0%)
Neurological	8 (13.3%)
Esophageal (reflux, motility disorders, esophagitis)	7 (11.7%)
Cervical osteophyte	5 (8.3%)
Presbyphagia	5 (8.3%)
Esophageal cancer	1 (1.7%)
Total	60 (100%)

All 60 patients completed the EAT-10 questionnaire at the initial appointment (baseline), and 51 patients (85%) completed the follow-up questionnaires after 30 days (median 31 days, range 24–59 days, IQR 30–32 days). At baseline, 59 patients (98.3%) completed the 15D and WHODAS 2.0, with 50 patients (83.3%) completing both follow-up assessments. Half of the patients completed the questionnaires electronically, and half used printed versions. No significant differences in age or gender were found between those who returned the follow-up questionnaires and those who did not.

### Changes in EAT-10 Total Scores

**Fig. 1:**
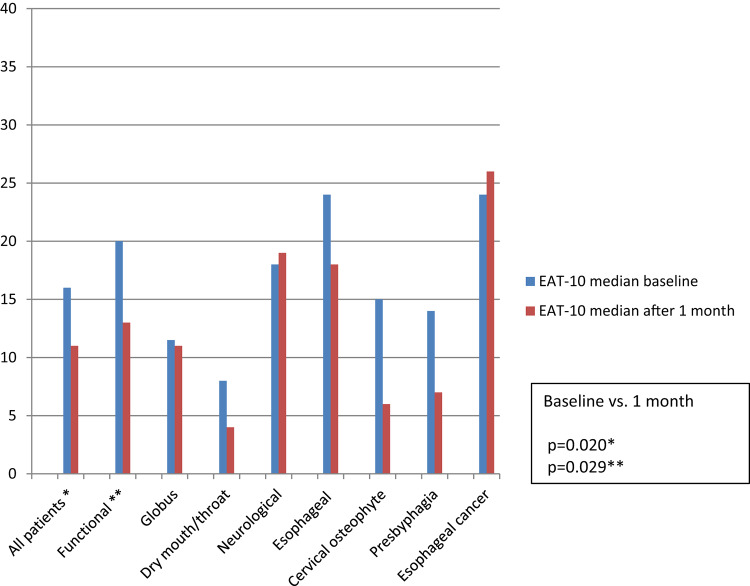
EAT-10 total score medians at baseline and one month after swallowing guidance, according to etiology of dysphagia. Statistically significant differences (*p* < 0.05) between total EAT-10 scores at baseline and one-month follow up are marked with an asterisk(s) (*)

The median EAT-10 scores at the initial appointment and at the one-month follow-up for all the dysphagia patients and specifically for those with functional dysphagia are presented in Table 2. EAT-10 scores decreased significantly following swallowing guidance (*p* = 0.020). Figure 1 shows median EAT-10 scores at baseline and after one month across different etiology groups. A significant decrease was observed in the functional dysphagia group (*p* = 0.029), while other etiology groups showed no significant changes, although medians tended to be lower after intervention, except in the neurological group and one case of esophageal cancer.

**Table 2:** EAT-10 total score medians from baseline and one-month follow up after swallowing guidance given to all dysphagia patients and to functional dysphagia patients

	EAT-10 median baseline (IQR), *n*	EAT-10 median after 1 month (IQR), *n*	*p*-value
All dysphagia patients	16 (8–24.75), *n* = 60	11 (5–21), *n* = 51	0.020*
Functional dysphagia patients	20 (16–31) *n* = 15	13 (9–27), *n* = 11	0.029*

Statistically significant differences (*p* < 0.05) between baseline and one-month follow up scores are marked with an asterisk (*)

### Changes in Subjective Symptoms

**Fig. 2:**
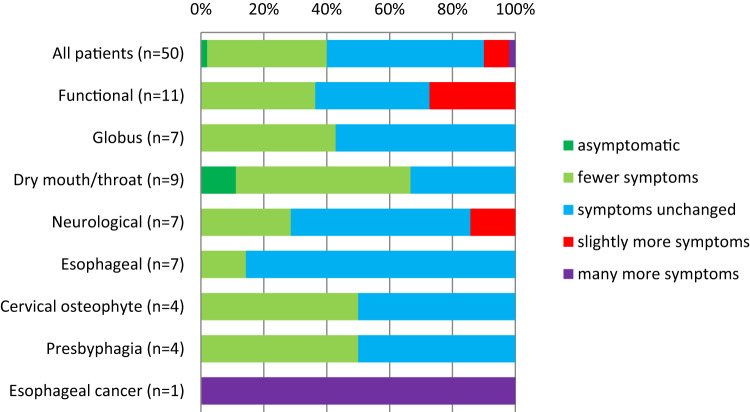
Subjective symptom change in one month after swallowing guidance according to etiology of dysphagia

Figure 2 illustrates patients’ subjective symptom changes one month after the initial appointment with a single intervention of FEES and swallowing guidance. In the entire cohort, 40% of patients reported symptom relief, while 50% felt symptoms remained unchanged. Most patients with dry mouth or throat (66.6%) reported fewer symptoms, while in other etiology groups, the majority reported no change. Notably, the only patient who reported significantly worse symptoms had malignant disease (esophageal cancer). Due to small group sizes, differences in subjective symptom change were not statistically significant. Additionally, the correlation between EAT-10 score changes and subjective symptom changes was weak (*p* = 0.060).

### Health-Related Quality of Life and Changes after Swallowing Guidance

**Fig. 3:**
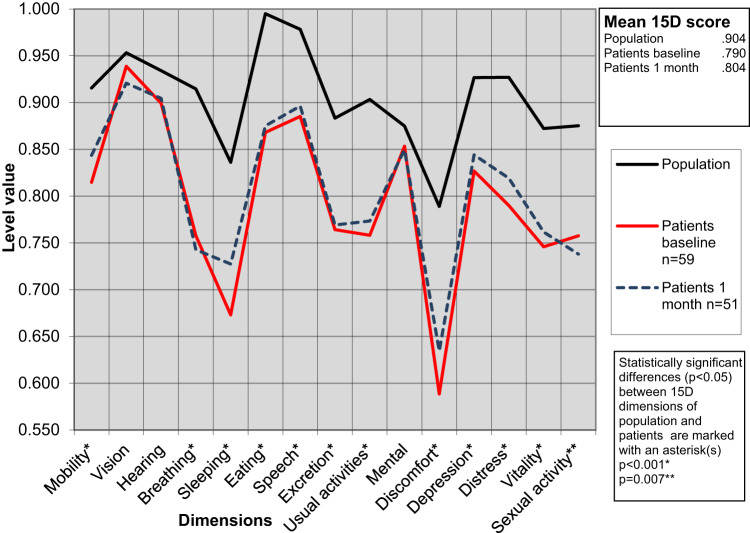
15D profiles of 59 dysphagia patients in comparison to those of general population, matched for gender and age, at baseline and one month after swallowing guidance

A comparison of the HRQoL profiles of dysphagia patients with an age- and gender-matched general population sample was conducted at baseline and after a one-month follow-up, as illustrated in Fig. 3. HRQoL scores differed significantly in overall 15D mean scores (*p* < 0.001, clinically important), and in multiple domains, including mobility (*p* = 0.001), breathing (*p* < 0.001), sleeping (*p* < 0.001), eating (*p* < 0.001), speech (*p* < 0.001), excretion (*p* < 0.001), usual activities (*p* < 0.001), discomfort and symptoms (*p* < 0.001), depression (*p* < 0.001), distress (*p* < 0.001), vitality (*p* < 0.001), and sexual activity (*p* = 0.007). No significant differences were observed in vision, hearing, and mental function. 15D mean scores were higher after a one-month follow-up in the functional, dry mouth/throat, cervical osteophyte, and globus diagnostic groups; however, the difference did not reach statistical significance (data not shown).

### Changes in Disability Scores

**Fig. 4:**
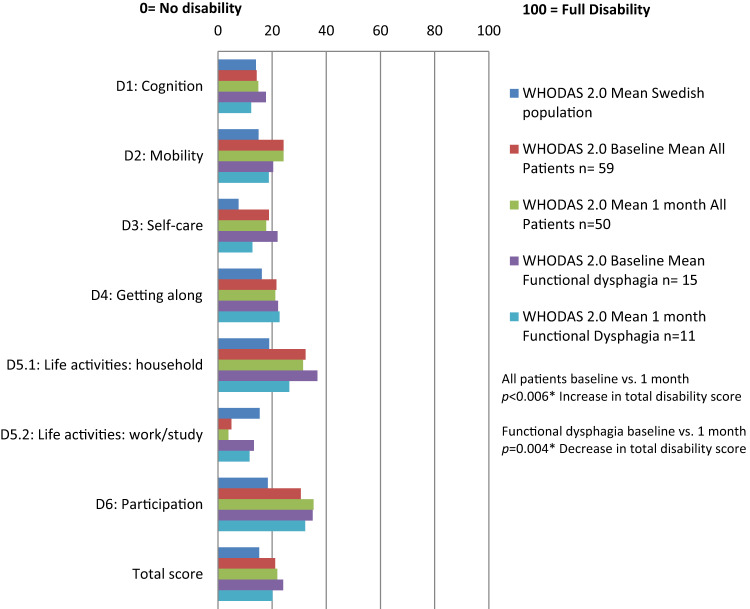
WHODAS 2.0 domain scores and summary scores in total patient cohort, and in functional dysphagia group at baseline and one-month follow-up after swallowing guidance. Reference to normal population scores in Swedish population [25]. Statistically significant differences (*p* < 0.05) between WHODAS 2.0 total scores at baseline and one-month follow-up are marked with an asterisk (*)

Figure 4 presents the WHODAS 2.0 domain and total scores at baseline and at one month for the entire patient cohort and the functional dysphagia group. Compared to normative values from the Swedish population, the patient cohort had higher disability scores in all domains except for work and study (Domain 5.2) [25]. Total WHODAS 2.0 scores and changes following swallowing guidance were statistically insignificant across the cohort. However, in the functional dysphagia group, there was a significant improvement in Domain 3 (self-care, *p* = 0.015), specifically item D3.3: ‘In the past 30 days, how much difficulty did you have in eating?’ (*p* = 0.020). No significant differences were observed in WHODAS 2.0 domains for other etiologies (data not shown).

### FEES Findings and sWST Test Results

The results of the sWST, categorized by dysphagia etiology, and the median EAT-10 scores are summarized in Table 3. Overall, 17 patients (28.3%) passed the sWST while 43 (71.3%) either required five or more swallows or coughed during the test. In the functional dysphagia group, three patients (20%) passed the sWST, and 12 (80%) failed due to small average drinking bolus size.

**Table 3:** Simplified water swallow test (sWST) results and EAT-10 total score medians at baseline and one month after swallowing guidance, according to etiology of dysphagia

Etiology of dysphagia	sWST passed	sWST failed	EAT-10 median baseline (IQR)	EAT-10 median after 1 month (IQR)
All *n* = 60	17 (28.3%)	43 (71.7%)	16 (8–24.75)	11 (5–21)
Functional *n* = 15	3 (20%)	12 (80%)	20 (16–31)	13 (9–27)
Globus *n* = 10	6 (60%)	4 (40%)	11.5 (7.25–16.75)	11 (7–15)
Dry mouth/throat *n* = 9	4 (44.4%)	5 (55.6%)	8 (3–22)	4 (2.5–9.5)
Neurological *n* = 8	0	8 (100%)	18 (10.25–23)	19 (9–27)
Esophageal (reflux, motility disorders, esophagitis) *n* = 7	2 (28.6%)	5 (71.4%)	24 (7–28)	18 (5–29)
Cervical osteophyte *n* = 5	2 (40%)	3 (60%)	15 (4.5–20)	6 (2.5–20)
Presbyphagia *n* = 5	0	5 (100%)	14 (8.5–21)	7 (2.5–16.75)
Esophageal cancer *n* = 1	0	1 (100%)	24	26

Statistically significant (*p* < 0.05) decrease in EAT-10 scores were found for all patients and in functional dysphagia group (*p* = 0.020, *p* = 0.029, respectively)

In the functional dysphagia group, 13 patients (86.7%) showed normal FEES results. One patient had minor vallecular saliva retention, and another showed abnormal pharyngeal wall movement but had normal neurological evaluations, so this case was classified as functional (data not shown).

No significant gender-based differences were found in EAT-10 score changes or subjective symptom changes from baseline to follow-up. Age did not correlate with changes in EAT-10 scores. Additionally, there were no significant differences in EAT-10 or symptom change outcomes between the two SLPs who provided the swallowing guidance.

## Discussion

For functional dysphagia patients, the one-time intervention of FEES with swallowing guidance and video explanation led to a statistically significant decrease in EAT-10 scores and the WHODAS 2.0 item score related to difficulty eating after the one-month follow-up. However, among the patients with other dysphagia etiologies, the changes in EAT-10 or WHODAS 2.0 scores were insignificant. Our results also indicate that dysphagia significantly impacts an individual’s HRQoL measured using the 15D in comparison to the normative values of the Finnish population. This result is in line with studies of other nationalities [1, 27, 28]. However, FEES with swallowing guidance did not significantly change the mean 15D scores after one-month follow-up.

One-fourth of the patients in our unselected dysphagia patient cohort were classified as having functional dysphagia, a prevalence consistent with our earlier prospective study [6]. We found a statistically significant decrease in the EAT-10 scores of the functional dysphagia group after the short one-time intervention of FEES with swallowing guidance. However, among the neurological patient group and in one case of esophageal cancer diagnosed after the outpatient appointment, no decrease was found in the EAT-10 scores, probably due to the progressive nature of these diseases. The EAT-10 total score medians suggested most patients benefited from the swallowing guidance, as indicated by decreased scores in the functional, dry mouth/throat, esophageal dysphagia, presbyphagia, and cervical osteophyte groups, though these changes were not statistically significant, likely due to small sample sizes. The 15D means after a one-month follow-up were also higher, indicating better HRQoL in the functional, dry mouth/throat, cervical osteophyte, and globus groups. However, statistical significance was not achieved due to the small sample size, as determined by a post hoc analysis.

On the other hand, when we asked for the patients’ subjective feelings of symptom change, the benefit of the swallowing guidance was not so evident. The majority of the patients felt that their symptoms remained unchanged after one-month follow-up. Only in the group of dry mouth/throat patients, 66.6% felt asymptomatic or that they had fewer symptoms after the intervention. This is perhaps due to concrete instructions to humidify and lubricate the mucosa. It is also notable that in this cohort, the only case of malignancy (esophageal cancer), had a high EAT-10 total score and the only considerable increase in subjective symptoms, underlining the fact that an increase in symptoms needs to be taken seriously.

Interestingly, the correlations between subjective symptom change and changes EAT-10 scores were weak. This could be due to the small sample size or to the personal response set, when some respondents prefer extreme answers creating higher variability in sum scores [29]. EAT-10 also assesses different aspects of dysphagia and the sum score may not represent the overall feeling of symptom change of the patient.

Globus patients were not excluded from this cohort because they were referred to phoniatrics due to dysphagia symptoms. However, at the initial appointment, their diagnosis was more precisely determined to be globus. Based on changes in the EAT-10, 15D and WHODAS 2.0 scores, globus patients did not appear to derive as clear a benefit from short swallowing guidance intervention as, for example, the functional dysphagia group after a one-month follow-up. This is likely because globus is not a true swallowing disorder, despite often being mistaken for one. In our experience, globus is primarily caused by muscle tension and stress, and its alleviation typically takes longer than one month [30, 31].

Functional symptoms are not uncommon nor are they a new phenomenon. Among the new referrals to a general practitioner, functional symptoms screening was as high as 25% or 35%, and in 20–25% of patients, these symptoms tend to be persistent or reappear over time [32–34]. This is in line with our observation among dysphagia patients in the present study as well as in our earlier study, in which 26.5% of dysphagia patients were classified as functional [6]. The treatment of functional symptoms can be challenging. Functional symptoms can develop after infections, injuries, medical conditions, stressful life events, or appear without a clear cause. As these symptoms continue, their connection to identifiable underlying physical issues often becomes less clear, complicating both diagnosis and treatment [3]. There are often maintaining factors that prolong the symptoms. These factors can be classified as physiological (autonomic dysfunction, sleep disorders, elevated sensitivity in the central nervous system, and imbalances in the hypothalamic-pituitary-adrenal axis), social (role confusion and ambiguity in medical diagnosis), cognitive (exaggerated interpretation of symptoms, fixation on symptoms, and discomfort with uncertainty), or behavioral (avoidance behaviors, extreme behaviors, and unhealthy sleep habits) [35]. Treatment for functional symptoms usually involves changing factors that can be modified, especially those related to thoughts and behaviors. A 2014 Cochrane review of non-pharmacological interventions of medically unexplained symptoms showed that cognitive behavioral therapy was more beneficial than other therapies, but the effect was not superior to enhanced care delivered by the individual’s physician [36]. In the present study, swallowing guidance seemed to especially help patients with functional dysphagia symptoms. As shown in other studies of functional symptoms, patients benefit good patient–doctor relationships and thorough validation of their symptoms [3, 7, 35, 37, 38]. Moreover, further examinations and referrals may only worsen the condition by reinforcing unhelpful illness behavior and symptom interpretation [38]. The FEES and swallowing guidance with video explanation can be valuable for assuring a patient that no severe malfunctions or pathologies are present and can help establish a good patient–doctor relationship.

A diagnosis of functional dysphagia requires thorough anamnesis, and some instrumental examination, such as the FEES, to rule out organic or neurological causes of dysphagia. Screening tools, such as WSTs, cannot be used to determine whether dysphagia is functional or organic. A study in the Netherlands found that most functional dysphagia patients used multiple swallows for the same bolus (piecemeal deglutition), considered a habitual coping strategy [39]. Similarly, in the present study, most functional dysphagia patients failed the sWST due to small drinking boluses, potentially indicative of functional dysphagia.

This prospective study of unselected dysphagia patients had some limitations. The number of patients in each of the etiology groups was small, and the probable effect of the swallowing guidance intervention remained unclear in those groups, although most of the patients seemed to benefit from the swallowing guidance, as shown by the decrease in the EAT-10 scores. The actual effect of the swallowing guidance would require case control studies and much larger patient samples. However, efficacy studies of different therapeutic interventions are difficult to carry out due to several confounding effects, small subpopulations, and unstandardized therapeutic protocol. In addition, the functional dysphagia group was heterogeneous, and the duration of the symptoms or the presence of other functional symptoms or neuropsychiatric comorbidities were not assessed. A one-month follow-up is also short, and longer follow-up might have shown longer-term effects of the swallowing guidance. However, our primary aim was to investigate the functional dysphagia group. This short intervention appeared to quickly alleviate fear and anxiety by validating and explaining the condition. Finally, globus patients were not excluded from the study although they are not really ‘true’ dysphagia patients.

Functional disorder needs to be detected and treated accordingly, and the sooner the diagnosis is achieved, the better the treatment outcomes [38]. Functional symptoms result in significant additional healthcare costs each year, like those associated with mental health conditions such as depression and anxiety disorders. These costs can be reduced through interventions that focus on both healthcare providers and patients, reducing both unnecessary examinations and referrals [40]. The brief, one-time swallowing guidance (a “mini-intervention”) we introduced is easy to implement and may benefit many dysphagia patients. It is also cost-effective, as it could serve as the sole diagnostic or therapeutic approach, particularly for functional dysphagia. Further investigations are warranted in the presence of alarming signs or worsening symptoms.

## Conclusions

Dysphagia significantly decreases patients’ HRQoL compared to the general population. A brief one-time intervention of FEES with swallowing guidance and video explanation could be particularly beneficial for patients with functional dysphagia, a commonly encountered condition.

## Data Availability

Data cannot be shared publicly because the ethical approval for this study applies of data on a group level, not an individual level. Therefore, data for individual cannot be made publicly available.
